# Herpes simplex virus 1 and the risk of dementia: a population-based study

**DOI:** 10.1038/s41598-021-87963-9

**Published:** 2021-04-22

**Authors:** Meghan J. Murphy, Lana Fani, M. Kamran Ikram, Mohsen Ghanbari, M. Arfan Ikram

**Affiliations:** 1 Department of Epidemiology, Erasmus MC-University Medical Center Rotterdam, PO Box 2040, 3000CA Rotterdam, The Netherlands; 2 Department of Neurology, Erasmus MC-University Medical Center Rotterdam, Rotterdam, The Netherlands

**Keywords:** Biomarkers, Diseases, Neurology

## Abstract

Herpes simplex virus 1 (HSV1) is a neuroinvasive virus capable of entering the brain which makes it a candidate pathogen for increasing risk of dementia. Previous studies are inconsistent in their findings regarding the link between HSV1 and dementia, therefore, we investigated how HSV1 relates to cognitive decline and dementia risk using data from a population-based study. We measured HSV1 immunoglobulin (IgG) antibodies in serum collected between 2002 and 2005 from participants of the Rotterdam Study. We used linear regression to determine HSV1 in relation to change in cognitive performance during 2 consecutive examination rounds on average 6.5 years apart. Next, we determined the association of HSV1 with risk of dementia (until 2016) using a Cox regression model. We repeated analyses for Alzheimer’s disease. All models were adjusted for age, sex, cardiovascular risk factors, and apolipoprotein E genotype. Of 1915 non-demented participants (mean age 71.3 years, 56.7% women), with an average follow-up time of 9.1 years, 244 participants developed dementia (of whom 203 Alzheimer’s disease). HSV1 seropositivity was associated with decline in global cognition (mean difference of HSV1 seropositive vs seronegative per standard deviation decrease in global cognition − 0.16; 95% confidence interval (95%CI), − 0.26; − 0.07), as well as separate cognitive domains, namely memory, information processing, and executive function, but not motor function. Finally, HSV1 seropositivity was not associated with risk of dementia (adjusted hazard ratio 1.18, 95% CI 0.83; 1.68), similar for Alzheimer’s disease. HSV1 is associated with cognitive decline but not with incident dementia in the general population. These data suggest HSV1 to be associated only with subtle cognitive disturbances but not with greater cognitive disorders that result in dementia.

## Introduction

Dementia is a problem that continues to rise as the elderly population increases, while the exact mechanisms of its cause are unknown. Immunity and inflammation have been implicated as possible pathways in the development of this disease^1^. A potential trigger for an immune response and inflammation is an infection, specifically, caused by herpes simplex virus 1 (HSV1). HSV1 is a neuroinvasive and neurotoxic virus capable of entering the brain via the peripheral nerves, and it is thus a candidate pathogen for increasing dementia risk^2,3^.

HSV1 is highly prevalent worldwide. Once infected, the virus remains latent in the body and can be reactivated throughout a person’s lifetime^2^. This constant presence and potential reactivation of the virus has been implicated in the immune response and subsequent development of Alzheimer’s disease^4^. The virus initiates an innate immune system response that causes the production of amyloid beta^1^. The accumulation of amyloid beta due to increased production subsequently causes neuroinflammation. The constant inflammation leads to damage to the brain cells (i.e. neurodegeneration) and eventually could result in dementia. Indeed, we have previously shown that an increase of various innate immune cells, in particular platelets, are associated with an increased risk of dementia in the general population^5^. Previous studies have looked at HSV1 and dementia, although mainly cross-sectionally with inconsistent findings^6^. Hence, there is still a gap in knowledge on how the virus affects the various stages of deteriorating cognition.

In this study, we determined the association of HSV1 with cognitive decline and incident dementia. We hypothesized that HSV1 is associated with poorer (global) cognition and subsequently an increased risk of dementia.

## Methods

### Study population

**Figure 1:**
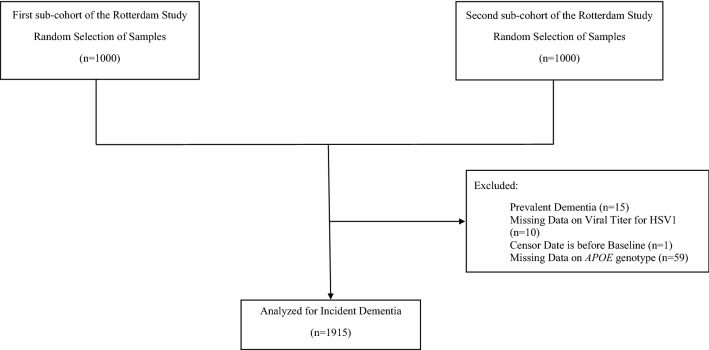
Flowchart for selection of participants for incident dementia analysis.

This study was embedded within the Rotterdam Study, a prospective population-based cohort study in Rotterdam, The Netherlands^7^. Within the study there are currently 4 cohorts; however, due to the availability of serum samples for HSV1 measurement this study used the first and second cohorts of the Rotterdam study. The first cohort began in 1990 and the second cohort began in 2000. Both cohorts started with participants of age 55 years or older at baseline. A total of 2000 randomly selected serum samples, 1000 from each cohort, were collected at examination rounds between 2002 and 2005. Baseline for this study was considered the ascertainment date of serum samples from participants (fourth visit for the first cohort, January 2002 through July 2004, and second visit for the second cohort, July 2002 and December 2005). Out of the 2000 participants, 10 were excluded due to missing HSV1 data because the measurement of antibodies titers was not possible from the plasma samples (e.g., no detectable antibodies or negative viral isolation) and 59 were excluded for missing Apolipoprotein E (*APOE*) carriership. Participants with prevalent dementia were also excluded; therefore, the final study group consisted of 1915 participants (Fig. 1).

The Rotterdam Study has been approved by the Medical Ethics Committee of the Erasmus MC (registration number MEC 02.1015) and by the Dutch Ministry of Health, Welfare and Sport (Population Screening Act [WBO], license number 1071272-159521-PG). The Rotterdam Study personal registration data collection is filed with the Erasmus MC Data Protection Officer under registration number EMC1712001. The Rotterdam Study has been entered into the Netherlands Trial Register (https://www.trialregister.nl) and into the WHO International Clinical Trials Registry Platform (https://www.who.int/ictrp/network/primary/en/) under the shared catalogue number NTR6831. In accordance with the Declaration of Helsinki, all participants provided written informed consent to participate in the study and to have their information obtained from treating physicians.

### Assessment of HSV1 antibodies

The serum samples were collected and centrifuged in EDTA treated containers. The aliquoted plasma was frozen at – 80 °C based on standard procedures. From the 2000 serum samples, chemiluminescence immunoassay (CLIA) was done to determine if immunoglobulin G antibodies (IgG) to HSV1 were present. The DiaSorin LIASION HSV1/2 IgG assay analyzer gave an index value ranging 0.5–30, based on the presence of the antibodies. Samples with an index value below 0.9 were considered negative for the virus. Index values ranging from 0.9 to 1.1 were considered equivocal^8^. Based on these ranges, a dichotomous variable for the virus was created. Values below 1.1 were defined as seronegative, while values of 1.1 and above were defined as seropositive for the HSV1 antibodies. No information was available on HSV immunoglobulin M antibodies (IgM) status.

### Assessment of global cognition and cognitive domains

Participants underwent a battery of cognition tests at the baseline examination visit as well as the next follow-up examination^9^. The tests included Mini Mental State Examination (MMSE), Word Learning Test (WLT immediate and WLT delayed recall), Letter-Digit Substitution Task (LDST), Verbal Fluency Task (VFT), Stroop Test (reading subtask, color naming subtask, and the color-word interference subtask), and Purdue Pegboard Task (PPB). For global cognition, we calculated the g-factor (general cognitive factor) as a standardized compound score using principal component analysis with LDST, Stroop color-word interference subtask, VFT, WLT delayed recall, and PPB sum of both hands. On each test, a higher score indicates a better performance apart from the Stroop test. Data on cognition was collected at baseline and at subsequent examination rounds between 2009 and 2011 to track cognitive decline.

### Assessment of incident dementia and Alzheimer’s disease

Screening for dementia was done at baseline and throughout the study via examination visits and digital medical records. Participants underwent multiple steps before being diagnosed with dementia. The first step was the MMSE and the Geriatric Mental Schedule (GMS). If participants screened positive in either test (MMSE < 26 or GMS > 0) then it was followed by an examination with the Cambridge Examination for Mental Disorders of the Elderly (CAMDEX) and, if believed to have dementia, consequent neuropsychological examinations^10^. Any participant who was unable to visit the center was followed via digital medical records through general practitioners and the Regional Institute for Outpatient Mental Health Care. The final diagnosis was made by a consensus panel in accordance with standard criteria (DSM-III-R) Alzheimer’s disease (NINCDS–ADRDA) and vascular dementia (NINDS-AIREN)^11,12^. Follow-up was until January 1, 2016 with 95.5% of participants. Participants were censored at date of dementia diagnosis, death, or loss to follow-up.

### Covariates

Potential confounding variables were chosen based on previous literature^10^. Data on sex, age, and prevalent diseases were obtained at each examination visit. Information on smoking, alcohol consumption, and education was collected through interview. Body mass index (BMI) was calculated by weight in kilograms divided by height in meters squared. We grouped blood pressure and cholesterol into two categorical covariates, hypertension and hypercholesterolemia, due to the large number of covariates. Measurement of blood pressure was done while participants were in a sitting position with a random-zero sphygmomanometer. The average was taken from two measurements. Hypertension was then found if blood pressure was over ≥ 140/90 mmHg or if the participant was taking blood pressure lowering medication. Cholesterol and high-density lipoprotein (HDL)-cholesterol were collected in serum through an automated enzymatic procedure, the Boehringer Mannheim System. Hypercholesterolemia was categorized as having high cholesterol levels (> 6.2 mmol/L) or use of lipid lowering medication. *APOE* genotype was found via serum sample. Additional information from the serum samples was collected on high sensitivity C-reactive protein (hs-CRP) and platelet count.

### Statistical analysis

The continuous values of HSV1 IgG antibodies were standardized. We used both the continuous and the dichotomous values of HSV1 antibodies in the models. All models were corrected for sub cohort, sex, and age at baseline. We additionally adjusted for BMI, smoking, alcohol consumption, education, hypercholesterolemia, hypertension, *APOE* genotype, coronary heart disease, diabetes mellitus, and stroke, all measured at baseline.

The Stroop reading subtask scores were log transformed, the Stroop color naming and color-word interference subtask scores were square root transformed. In addition, the Stroop subtask scores were inverted by multiplying with − 1 to better match the other cognitive tests. All cognitive test results were standardized. Linear regression analysis was done for each cognition test performed during the fifth examination visit as outcome variable and the baseline (fourth examination visit) cognition test scores were adjusted for in the model. First the g-factor and MMSE were analyzed after which we explored each test separately.

A Cox regression analysis was performed to study the association between HSV1 and incident dementia and Alzheimer’s disease (AD). For incident dementia, we performed a sensitivity analysis using varying cutoffs for seroprevalence to assess effects of varying antibody levels (index value of cutoffs for seroprevalence: 0.7–1.4 with 0.1 increment increase). In addition, we performed stratification to assess effect modification and calculated multiplicative interaction by age (10-year intervals), sex, hs-CRP (threshold: > 2 mg/L), *APOE*-ε4 carrier status, and platelet levels (based on the median; threshold: > 252×${10}^{9}$ per liter)^5^. Interactions by hs-CRP and platelet levels were analyzed to check whether a higher innate immunity status affected the link of HSV1 with dementia compared to having a lower innate immunity status.

The assumptions for linear regression and Cox regression were checked for all analyses. Missing data was imputed 5 times using multiple imputation by chained equations (MICE) using the outcome and covariates as predictors to impute missing covariates, pooling the 5 imputations. All analyses were completed using IBM SPSS Statistics version 25.0 (IBM Corp., Armonk, NY, USA).

## Results

The baseline characteristics of the study population are in Table 1. The mean age at baseline was 71.3 years ($\pm$ 7.5). Of 1915 participants, 1518 were HSV1-seropositive (79.3%).

**Table 1:** Baseline Characteristics of the Study Population.

Characteristics	Cohort undergoing cognitive examination* (N = 1249)	Dementia-free cohort (N = 1915)
Females	719 (57.6%)	1086 (56.7%)
Age, years	69.0 ± 6.3	71.3 ± 7.5
**Cohort**		
First	671 (45.7%)	928 (48.5%)
Second	678 (54.3%)	900 (50.3%)
**Education**		
Primary education	95 (7.7%)	203 (10.7%)
Lower/intermediate general education	546 (44.1%)	849 (44.8%)
Intermediate vocational education	382 (30.9%)	564 (29.7%)
Higher vocational education	214 (17.3%)	281 (14.8%)
**Apolipoprotein E4ε carriership**		
Non-carrier (ε2/ε2, ε2/ε3 or ε3/ε3)	918 (73.5%)	1398 (73.0%)
Carrier (ε4/ε4, ε3/ε4 or ε2/ε4)	331 (26.5%)	517 (27.0%)
**Smoking**		
Current	171 (13.9%)	270 (14.3%)
Former	692 (56.1%)	1053 (55.7%)
Never	370 (30.0%)	568 (30.0%)
Body mass index, kg/m^2^	27.7 ± 3.9	27.6 ± 4.1
Hypertension	913 (73.1%)	1485 (77.5%)
Hypercholesterolemia	607 (51.2%)	900 (47.2%)
Alcohol consumption	12.7 ± 14.1	12.1 ± 14.7
History of coronary heart disease	108 (8.7%)	206 (10.9%)
Diabetes mellitus type 2	142 (11.6%)	279 (15.2%)
History of stroke	35 (2.8%)	78 (4.1%)

*N* number of participants included in study. Data presented as mean (standard deviation) for continuous variables and number (percentages) for categorical variables. Data represent original data without imputed values.

*Participants underwent multiple cognitive tests.

Of 1900 participants (99.6%) who underwent detailed cognitive assessment at baseline, 1249 (65.4%) had repeated assessment at follow-up (mean interval 6.5 years). Within HSV1 seropositive participants, the mean difference in g-factor score was lower by 0.16 (95% confidence interval [95% CI]: − 0.26; − 0.07) compared to HSV1 seronegative participants. Per standard deviation (SD) increase in HSV1 antibody titer the mean difference in g factor [z score] was -0.04 (− 0.08; 0.002). For MMSE, the mean difference in z score for HSV1 seropositive participants compared to seronegative participants was lower by 0.12 (− 0.24; 0.002) compared to seronegative participants and per SD increase in HSV1 antibody titer − 0.06 (− 0.11; − 0.01).

HSV1 was significantly associated with several cognitive tests. WLT delayed had the largest difference with a z score mean difference of − 0.12 (− 0.24; 0.004) for HSV1 seropositive compared to HSV1 seronegative. Seropositive HSV1 was associated with decreased z scores in LDST. HSV1 seropositive was significant in both the VFT (− 0.13, − 0.24; − 0.03) and the LDST (− 0.12, − 0.21; − 0.04) compared to seronegative participants. Results were similar for HSV1 antibody titer. Neither HSV1 seropositivity nor HSV1 antibody titer showed a significant decrease in the PPB Sum test (Table 2).

**Table 2:** Association of serum HSV1 seroprevalence and titer with cognitive decline in the Rotterdam study.

	Mean change in cognition in standard deviation (95% CI)									
	G-factor	MMSE	WLT Immediate	WLT Delayed	Stroop: reading	Stroop: color naming	Stroop: color-word	LDST	VFT	PPB
	n = 901	n = 1249	n = 1091	n = 1091	n = 1154	n = 1152	n = 1145	n = 1159	n = 1193	n = 1029
HSV1 serum seroprevalence	− 0.16 (− 0.26; − 0.07)	− 0.12 (− 0.24; 0.002)	− 0.03 (− 0.14; 0.09)	− 0.12 (− 0.24; 0.004)	− 0.10 (− 0.21; 0.01)	− 0.08 (− 0.19; 0.04)	− 0.11 (− 0.23; 0.01)	− 0.12 (− 0.21; − 0.04)	− 0.13 (− 0.24; − 0.03)	− 0.09 (− 0.19; 0.01)
HSV1 serum IgG antibody titer	− 0.04 (− 0.08; 0.002)	− 0.06 (− 0.11; − 0.01)	− 0.02 (− 0.07; 0.03)	− 0.07 (− 0.12; − 0.02)	− 0.04 (− 0.08; 0.01)	− 0.02 (− 0.06; 0.03)	− 0.02 (− 0.07; 0.03)	− 0.04 (− 0.08; − 0.01)	− 0.05 (− 0.10; − 0.01)	− 0.02 (− 0.06; 0.03)

The Stroop subtask scores were inverted to better match the other cognitive tests. In addition, the Stroop reading subtask scores were log transformed, the Stroop color naming and color-word interference subtask scores were square root transformed. All cognitive test results were standardized. Linear regression analysis was done for each cognition test performed during the fifth examination visit as outcome variable and the baseline (fourth examination visit) cognition test scores were adjusted for in the model.

Linear Regression Model adjusted for sub cohort, sex, age, body mass index, smoking, alcohol consumption, education, hypertension, hypercholesterolemia, apolipoprotein E carrier status, coronary heart disease, diabetes mellitus, and stroke.

*MMSE* Mini Mental State Examination, *WLT* Word Learning Test, *LDST* Letter-Digit Substitution, *VFT* Verbal Fluency Test, *PPB* Purdue Pegboard Test: Sum of Both Hands, *CI* confidence interval, *n* number of subjects, *IgG* immunoglobulin G.

During an average follow-up time of 9.1 years ($\pm$ 3.4), 244 participants were diagnosed with dementia, 203 of whom were diagnosed with Alzheimer’s disease. HSV1 seropositivity was not associated with a higher risk of all-cause dementia (adjusted hazard ratio [HR] of 1.18 (95% CI 0.83; 1.68)). Per SD increase of antibody titer of HSV1, the HR was 1.03 (0.90; 1.17). For Alzheimer’s disease, the results were similar for HSV1 seropositive and HSV1 antibody titer (1.13, 95% CI 0.77; 1.66 and 1.05, 95% CI 0.90; 1.21, respectively) (Table 3).

**Table 3:** Association of serum HSV1 seroprevalence and titer with Incident Dementia in the Rotterdam Study.

	Hazard ratio of dementia (95% CI)			Hazard ratio of Alzheimer's disease (95% CI)		
	n/N	Model I	Model II	n/N	Model I	Model II
HSV1 serum IgG seronegative	38/397	Reference	Reference	32/397	Reference	Reference
HSV1 serum IgG seropositive	206/1518	1.21 (0.86; 1.72)	1.18 (0.83; 1.68)	171/1518	1.18 (0.80; 1.72)	1.13 (0.77; 1.66)
HSV1 serum IgG antibody titer per standard deviation increase	244/1915	1.06 (0.93; 1.20)	1.03 (0.90; 1.17)	203/1915	1.08 (0.94; 1.25)	1.05 (0.90; 1.21)

Cox Model I, adjusted for sub cohort, sex, and age. Cox Model II, adjusted for sub cohort, sex, age, body mass index, smoking, alcohol consumption, education, hypertension, hypercholesterolemia, apolipoprotein carrier status, coronary heart disease, diabetes mellitus, and stroke.

*HR* hazard ratio, *CI* confidence interval, *n* number of cases, *N* number of subjects, *IgG* immunoglobulin G.

**Figure 2:**
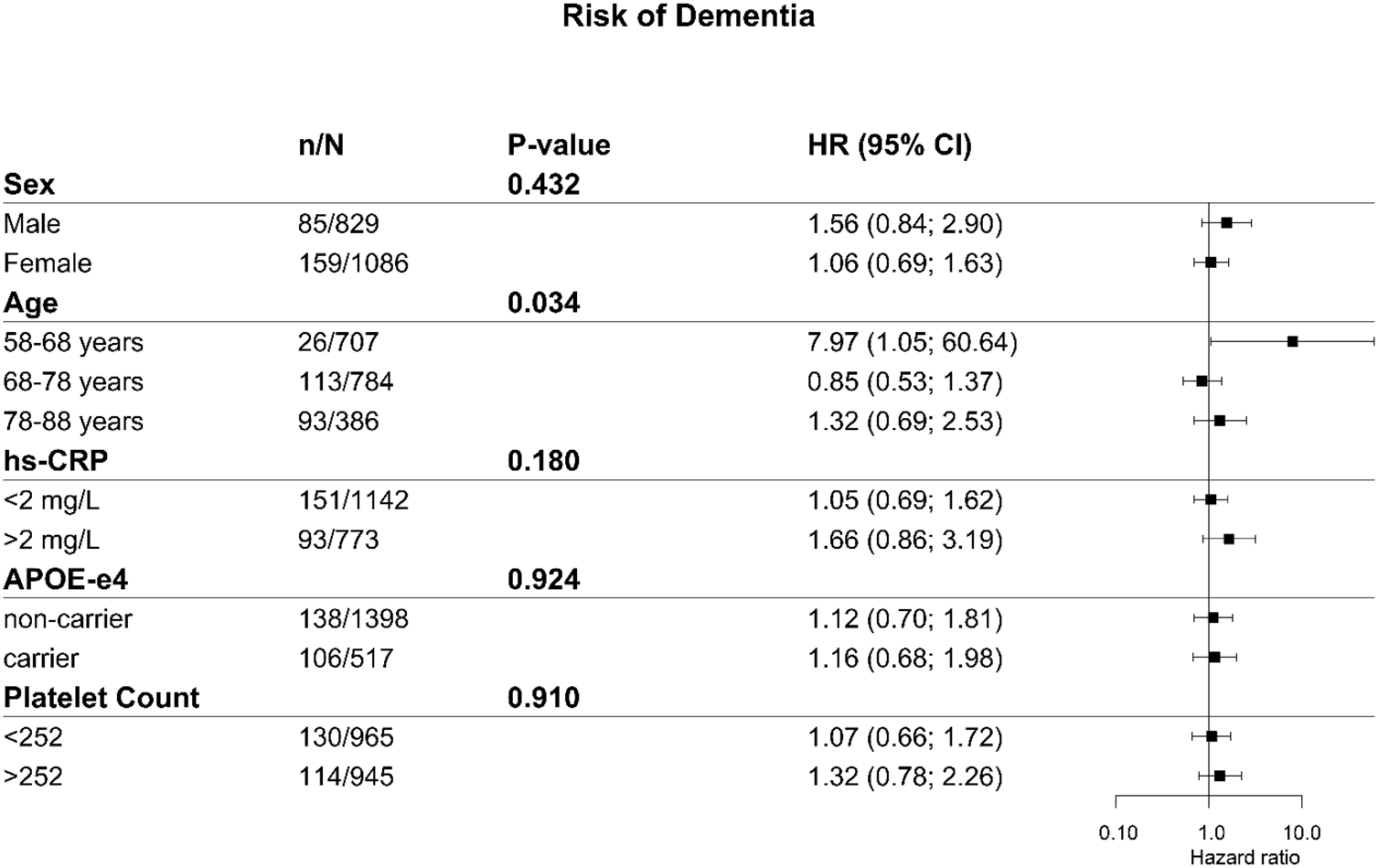
Stratification of various factors for the relation of HSV1 seroprevalence to the risk of dementia. n/N represents the number of dementia cases. P value is for the interaction term of the grouping variable and HSV1 seroprevalence. *HR* hazard ratio of incident dementia, *CI* confidence interval.

The different cut-off levels for HSV1 seroprevalence showed similar results (Figure S1). Seropositive males had a larger hazard ratio of dementia than seropositive females (1.56, 95% CI 0.84; 2.90 and 1.06, 95% CI 0.69; 1.63, respectively), although the multiplicative interaction between sex and HSV1 seropositive was not significant. The hazard ratio for HSV1 seroprevalent dementia cases varied widely between age groups, with the interaction term for age and HSV1 seroprevalence being significant (p = 0.034). The highest age category, 88–98 years old, was not analyzed due to lack of power. The group with higher hs-CRP levels (> 2 mg/L) had a higher hazard ratio for seroprevalence (HR 1.66) compared to the group with lower hs-CRP levels (HR 1.05), with the multiplicative interaction between the virus and CRP being borderline significant (p = 0.180). The group with higher platelet levels (> 252×${10}^{9}$ per liter), had a higher HR of 1.32 (95% CI 0.78; 2.26) compared to lower platelet levels for the seropositive group (HR 1.07), however we observed no significant interaction. *APOE*-ε4 carriers had no effect change for HSV1 seropositivity (Fig. 2). All assumptions were met for all analyses.

## Discussion

In this study, cognitive decline was more pronounced in HSV1 seropositive compared to seronegative participants in global cognition and the cognitive domains of memory, information processing and executive function^13^. There was no association between HSV1 and the development of dementia.

The current literature mainly focuses on the final stage of deteriorated cognition: dementia^6^ and only few studies have investigated cognition^12,13^. In the studies that found an association between declined cognition and HSV, it was only with high infectious burden, i.e. multiple viruses (a combination of HSV1, HSV2, and Cytomegalovirus)^14^ or in carriers of *APOE*-ε4^15^. One study found no association between HSV1 and cognitive decline^16^.

Previous studies found conflicting results regarding the association between HSV1 and dementia^6^. Two previous cohort studies varied from our cohort in having a shorter follow-up time (the longest being 4 years), which makes these studies more prone to reverse causation^17,18^. In a Taiwanese retrospective cohort study, they found an association, but all the HSV cases were not determined via serology but rather based on a minimum number of medical visits for HSV symptoms. Therefore, it is most likely that active or reactivated HSV infections were included in the study which may have resulted in stronger effects than our study on latent HSV1 infections^19^. Two previous studies only saw the association in *APOE*-ε4 carriers^20,21^, unlike our study. In a nested case–control study, the presence of anti-HSV IgG and IgM antibodies did not increase the risk of AD significantly^22^. In one of the few longitudinal studies on the subject, an association was found only with HSV1 IgM and AD but not with HSV1 IgG antibodies and AD (HR: 0.99, 95% CI 0.57; 1.72)^23^ which is consistent with our study. Another study looking at HSV IgG antibodies and dementia revealed a HR of 1.67 (95% CI 0.75; 3.73)^24^, yet both of these studies failed to differentiate between HSV1 and HSV2 antibodies. A third prospective cohort study studying the same association found a HR of 0.77 (95% CI 0.58; 1.04) for HSV1 specific IgG antibodies^25^. It is possible that the presence of HSV1 IgG antibodies does not lead to constant inflammation; therefore, the latent virus is not associated with greater cognitive disturbances. Since our study comprised the general population, a high prevalence of latent HSV1 was expected as opposed to a reactivated infection.

A potential mechanism for an HSV1 infection to affect cognition is via a temporary production of amyloid beta to repress the infection. This leads to short-lived inflammation which affects cognition according to the recent antimicrobial protection hypothesis of AD^1^. Once the infection and subsequent inflammation are gone, cognition is no longer impaired. However, when reactivation occurs, we would expect an association between HSV1 and dementia due to a chronic inflammatory process.

In our study, the HSV1 infection as measured by IgG antibodies (latent infection) are associated with a subtle decrease in global cognition over time. Due to lack of information on cognition tests at later examination dates (after 2011) in our study, we do not know whether cognition stayed at the new decreased level or if it returned to the previous level measured at baseline. However, our results suggest that it did not decrease further; otherwise, we would have observed an association between HSV1 and dementia. The combination of multiple neurotoxic viruses may be required to cause permanent damage to cognitive function, therefore HSV1 alone is not enough to see a significant effect.

A major strength of this study was the availability of prospectively collected data and the length of follow-up time. In addition, we were able to analyze both the subtle (i.e. cognition) and large changes in cognition (i.e. dementia) in one study.

It is also important to consider the limitations of this study. A major one is the availability of only HSV1 IgG antibodies which only gave us information on infection prior to baseline. Without data on the HSV1 IgM, we were unable to tell whether the participants had an active HSV1 infection at baseline. Since the reactivation of the virus is the proposed pathway to deteriorating cognition, we would need to investigate HSV1 IgM antibodies and each stage of deteriorating cognition to gain a better understanding. Another limitation is the lack of generalizability as the Rotterdam Study cohort predominantly consists of Caucasian participants living in a middle-income area in Rotterdam. A final limitation is the lack of antiviral medication data within our study, since it could be a potential modifying factor in the association between HSV1 and dementia.

In conclusion, HSV1 is associated with a decrease in global cognition and cognitive domains, but not with dementia risk in the general population. These findings suggest that HSV1 IgG antibodies may lead to subtle cognitive disturbances but not greater cognitive disorders.

## Supplementary Information

Supplementary Information .
