# Supplementary material for: Herpes simplex virus 1 and the risk of dementia: a population-based study
Source: Sci Rep. 2021 Apr 22;11:8691. doi: 10.1038/s41598-021-87963-9 (PMC8062537; doi:10.1038/s41598-021-87963-9)
Supplement: Supplementary file 1 — Supplementary Information . [file 41598_2021_87963_MOESM1_ESM.pdf]

SUPPLEMENTS

Herpes Simplex Virus 1 and the Risk of Dementia: A Population-Based Study

Meghan Murphy MSc[a,±], Lana Fani MD[a,±], M. Kamran Ikram MD PhD[a,b], Mohsen Ghanbari MD PhD[a], M. Arfan Ikram MD PhD[a,*]

[a]Department of Epidemiology, Erasmus MC - University Medical Center Rotterdam, Rotterdam, the Netherlands

[b]Department of Neurology, Erasmus MC - University Medical Center Rotterdam, Rotterdam, the Netherlands

[±]These authors contributed equally

*Corresponding author: M. Arfan Ikram, MD PhD

Table S1. Mean Cognitive Scores at Baseline and Follow-up

Cognitive Test Score	Baseline Test			Follow-Up Test			
	N	Mean	Standard deviation	N	Mean	Standard deviation	Cognitive Domain
G-factor	901	-1.7	1	901	0.0	1.0	Global Cognition
Mini-Mental State Exam	1249	27.5	2.6	1249	27.4	2.8	Global Cognition
Word Learning Test: Immediate Recall	1091	6.8	2.1	1126	7.2	2.1	Memory
Word Learning Test: Delayed Recall	1091	6.5	2.8	1126	6.8	2.9	Memory
Stroop Test: Reading	1154	18.7	4.7	1199	18.3	4.1	Information Processing
Stroop Test: Color Naming	1152	24.9	6.3	1197	25.1	6.1	Information Processing
Letter-Digit Substitution Test	1159	27.2	7.1	1203	26.6	7.1	Information Processing, Executive Function
Stroop Test: Color-Word	1145	60.4	31.6	1190	60.0	27.2	Executive Function
Verbal Fluency Test	1193	20.7	5.4	1238	21.0	5.7	Executive Function
Purdue Pegboard Test: Sum of Both Hands	1029	33.5	5.2	1071	32.6	5.0	Motor Function

Note: n/N represents the number of individuals that underwent the cognitive tests.

Figure S1. Sensitivity Analysis of HSV1 Seroprevalence with Risk of Dementia using Different Seroprevalence Cut-offs

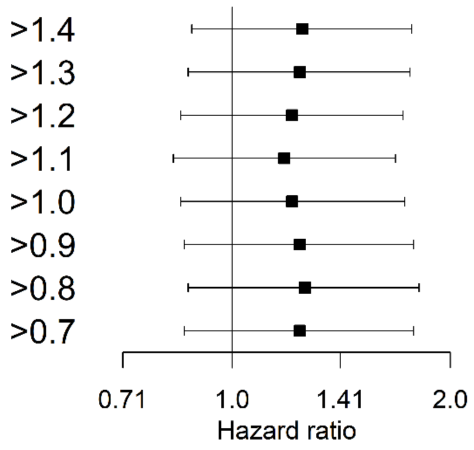

Note: Each row represents the hazard ratio of dementia for each index cut-off for HSV1 seroprevalence.
